# Evaluation of a modified triple‐combination anesthesia using dexmedetomidine in mice

**DOI:** 10.1002/ame2.70098

**Published:** 2025-11-18

**Authors:** Masaki Watanabe, Ryosuke Nakanishi, Tomoki Omori, Takeru Sasaki, Atsushi Asano, Nobuya Sasaki

**Affiliations:** ^1^ Laboratory of Laboratory Animal Science and Medicine, School of Veterinary Medicine Kitasato University Towada Japan; ^2^ Laboratory of Laboratory Animal Science, Joint Faculty of Veterinary Medicine Kagoshima University Kagoshima Japan

**Keywords:** anesthesia refinement, dexmedetomidine, mouse strains, thermoregulatory recovery, triple‐combination anesthesia

## Abstract

**Background:**

Ketamine is a widely used anesthetic in animal research, but its use is strictly regulated in several countries, including Japan and China. As an alternative, the medetomidine‐midazolam‐butorphanol (MMB) combination is commonly used in Japan. However, medetomidine is a racemic mixture containing the inactive R‐enantiomer, which may reduce anesthetic predictability and safety.

**Objective:**

The aim of the study was to evaluate the efficacy and safety of a modified anesthetic combination (dMMB), in which dexmedetomidine replaces medetomidine, across three commonly used mouse strains (ICR, C57BL/6, BALB/c).

**Methods:**

Male and female mice were administered either MMB or dMMB subcutaneously. Anesthetic depth, recovery profiles, heart rate, SpO_2_, body temperature, ocular opacity, and blood glucose levels were assessed. Atipamezole was used to reverse anesthesia, and thermoregulatory recovery was monitored postinjection.

**Results:**

dMMB produced similar anesthetic depth to MMB, with faster and more consistent recovery, particularly in males. Body temperature recovery was significantly enhanced in dMMB‐treated B6 males. No significant differences in side effects (ocular opacity or blood glucose levels) were observed between protocols, though strain‐specific glucose elevations were noted in dMMB‐treated males.

**Conclusion:**

dMMB is a safe, effective, and ketamine‐free injectable anesthetic protocol, offering advantages in recovery and thermoregulation. It may be a valuable alternative in research settings where ketamine is restricted and medetomidine may become unavailable.

## Graphical abstract

Development of a modified ketamine‐free anesthetic (dMMB) using dexmedetomidine. To improve the safety and recovery profile of conventional MMB, we developed dMMB by replacing medetomidine with dexmedetomidine. This ketamine‐free formulation showed comparable anesthetic depth and enhanced thermoregulation, avoiding unnecessary isomers for safer use.

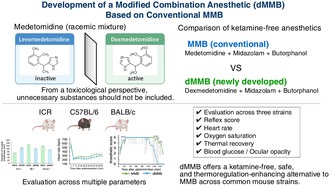

## 1 INTRODUCTION

Anesthetics are essential in animal‐based research not only for minimizing animal distress but also for ensuring scientific validity. Among various approaches, balanced anesthesia, which combines multiple agents to achieve an optimal mix of sedation, analgesia, and muscle relaxation, is widely recognized as a method that enables safe and effective anesthetic management due to complementary pharmacological actions and reduced side effects.^1^, ^2^

Globally, the combination of ketamine and xylazine is commonly used. However, in Japan, ketamine is classified as a controlled substance, and its use is strictly regulated. Consequently, an alternative triple‐combination anesthesia comprising medetomidine (Med), midazolam (Mid), and butorphanol (Bu), known as MMB anesthesia, is widely employed.^3^ Additionally, in several countries, including China, the use of ketamine is strictly regulated due to concerns over abuse. Since its designation as a Class I controlled substance in China in 2004, researchers have faced increasing restrictions on its acquisition and use.^4^ As a result, the development of ketamine‐free anesthetic protocols has become a practical necessity in Chinese laboratory settings.

Med, a component of MMB, exerts sedative and analgesic effects via α_2_‐adrenergic receptor agonism.^5^ However, its use is associated with cardiovascular side effects, such as bradycardia, cardiac depression, and hypothermia.^6^ Furthermore, Med is a racemic mixture containing equal parts of the pharmacologically active S‐enantiomer (dexmedetomidine: Dex) and the largely inactive R‐enantiomer (levomedetomidine: Levo), which may even counteract desired effects via α₁‐receptor stimulation.^7^, ^8^ The presence of this inactive isomer not only reduces the predictability of anesthetic effects but also raises toxicological concerns. To address these issues, Dex—a formulation consisting solely of the active S‐enantiomer—was developed. Dex exhibits high α_2_‐receptor selectivity and a favorable safety profile; consequently, it is now widely used in human medicine.^9^ In the field of veterinary medicine, efforts are underway to transition toward safer anesthetic protocols informed by findings from human clinical use.^10^ Accordingly, Dex is gaining attention in both laboratory animal anesthesia and companion animal practice. Additionally, atipamezole (Atip), a selective α_2_‐adrenergic receptor antagonist, is commonly used to reverse the effects of Med or Dex and promote rapid recovery from anesthesia. By quickly counteracting α_2_‐agonist effects, Atip shortens the duration of anesthesia and facilitates thermoregulatory recovery.^11^ This is particularly important in small laboratory animals, where rapid and safe postoperative recovery is essential for both scientific validity and animal welfare.

On the contrary, physiological and genetic differences among mouse strains are known to influence their sensitivity and responsiveness to anesthetic agents. The C57BL/6 (B6) strain, in particular, is the most widely used inbred mouse strain and is considered a “gold standard” in a wide range of research fields, including neuroscience, immunology, genetics, infectious diseases, cancer research, and toxicology.^12^ Due to its well‐defined genetic background and high compatibility with genetically modified strains, B6 mice also serve as a valuable reference model for evaluating anesthetic responses.^13^, ^14^, ^15^ The BALB/c (BALB) strain, characterized by a Th2‐skewed immune response, is frequently used in studies of allergy, inflammation, and host defense against infection.^16^ As reported previously, BALB mice showed a slightly earlier recovery from anesthesia compared to B6 mice despite no statistically significant differences in anesthetic duration.^17^ This suggests that BALB mice may serve as a useful comparative strain in anesthetic studies. Owing to their outbred background, ICR mice are widely used in toxicology, pharmacology, and reproductive studies^18^ and are considered suitable for evaluating population‐level variation in pharmacological effects. In fact, strain‐dependent differences in anesthetic responses have been reported, including studies directly comparing ICR mice with other strains.^19^ Thus, comparing anesthetic efficacy across these three strains—each with distinct genetic and physiological backgrounds—provides important insights for improving the external validity and reproducibility of experimental findings.

In this study, we formulated a Dex‐based triple‐combination anesthetic (dMMB: Dex, Mid, and Bu) and evaluated its effects in three mouse strains: B6, BALB, and ICR. We assessed anesthesia duration, reflex scores, heart rate, and peripheral oxygen saturation (SpO_2_). In addition, we examined the recovery kinetics following administration of Atip, with a particular focus on body temperature restoration. As part of the side effect evaluation, ocular opacity was visually inspected, and blood glucose levels were measured. The aim was to compare dMMB with the conventional MMB protocol in terms of efficacy and safety, as well as to characterize inter‐strain differences in anesthetic responsiveness.

## 2 MATERIALS AND METHODS

### 2.1 Ethical statement

This study adhered to the Kitasato University Regulations for the Care and Use of Laboratory Animals and received approval from the president of Kitasato University after undergoing assessment by the Institutional Animal Care and Use Committee (approval no.: 24‐097).

### 2.2 Animals

All experiments were conducted using 8–10‐week‐old mice of the following strains: Jcl:ICR (ICR), C57BL/6JJcl (B6), and BALB/cAJcl (BALB). The average body weights of male mice were 37.25 ± 1.89 g for ICR, 18.7 ± 1.86 g for B6, and 27.58 ± 1.32 g for BALB. Female mice weighed 28.94 ± 1.74 g for ICR, 19.73 ± 0.90 g for B6, and 20.70 ± 1.74 g for BALB. These differences in body weight reflect well‐documented phenotypic characteristics of each strain, as mice were standardized by age (8–10 weeks) rather than weight.^20^ Body weight values are expressed as mean ± standard deviation (SD). All animals were obtained from CLEA Japan (Tokyo, Japan) and were allowed to acclimate for 1 week prior to experimentation.^21^ The animal facility was maintained under controlled conditions, with a constant temperature of 22 ± 2℃, relative humidity of 40%–60%, and a 12‐h light/dark cycle. Animals were provided with standard laboratory chow (CE‐2; CLEA Japan) and tap water ad libitum.

### 2.3 Anesthetic drugs

The anesthetic agents used in this study were Med hydrochloride (Dorbene Vet, Kyoritsu Seiyaku Co., Tokyo, Japan), Dex hydrochloride (dexmedetomidine Sandoz, Sandoz Japan Co., Ltd., Tokyo, Japan), Mid hydrochloride (midazolam Sandoz, Sandoz Japan Co., Ltd., Tokyo, Japan), and Bu tartrate (Vetorphale, Meiji Seika Pharma Co., Ltd., Tokyo, Japan). All agents were commercially available as injectable solutions. The administration dosage of Med was standardized to 0.5 mg/kg, considering information from previous reports, the depth of surgical anesthesia, and strain variations, to facilitate comparison between the two mixed anesthetics.^22^ The dosage of Dex in the dMMB combination was determined based on the fact that Med is a racemic mixture containing only 50% of the pharmacologically active Dex. Therefore, the Dex dose was set at 0.25 mg/kg to match the active component equivalent of 0.5 mg/kg Med used in the MMB combination. This dosage falls within the range previously reported to induce effective and safe sedation in mice.^23^ The dosage of Mid was set at 4 mg/kg, in line with previous reports.^24^, ^25^, ^26^ Furthermore, the concentration of Bu was standardized to 5 mg/kg, in alignment with previous studies.^25^ The specific combinations and dosages of these drugs in the study are detailed in Table 1. For drug administration, each anesthetic mixture was diluted with normal saline to achieve a final volume of 0.01 mL per gram of body weight (e.g., for a 25‐g mouse, inject 250 μL). Intraperitoneal injection, although commonly used, is known to have a relatively high failure rate due to the risk of misdelivery into visceral organs, adipose tissue, or the subcutaneous space, resulting in variable anesthetic outcomes.^27^, ^28^ To minimize such inconsistencies, this study used subcutaneous injection at the dorsocervical region as the route of administration. The anesthetic agents were delivered using sterile disposable plastic syringes fitted with 29‐g needles (Myjector, Terumo Corp., Tokyo, Japan).

**TABLE 1:** Composition and dosage of conventional MMB and modified dMMB anesthesia in mice.

MMB	Medetomidine (Med)	Midazolam (Mid)	Butorphanol (Bu)	Saline	Total
Product conc. (mg/mL)	1	5	5	–	–
Agent dose (mg/kg)	0.5	4	5	–	–
Example (μL)	50	80	100	770	1000
Example for 25‐g mouse (μL)	12.5	20	25	192.5	250

dMMB	Dexmedetomidine (Dex)	Midazolam (Mid)	Butorphanol (Bu)	Saline	Total
Product conc. (mg/mL)	0.1	5	5	–	–
Agent dose (mg/kg)	0.25	4	5	–	–
Example (μL)	250	80	100	570	1000
Example for 25‐g mouse (μL)	62.5	20	25	142.5	250

*Note*: The table summarizes the formulation details for the conventional MMB (medetomidine, midazolam, and butorphanol) and modified dMMB (dexmedetomidine, midazolam, and butorphanol) anesthetic mixtures. Product concentrations (mg/mL), agent doses (mg/kg), and an example of preparation volume (μL) for a total volume of 1000 μL are shown for each component. Saline was added to adjust the final volume. The “example for a 25‐g mouse (μL)” indicates the injection volume calculated for a 25‐g mouse based on a final injection volume of 0.01 mL/g body weight. Saline was added to adjust the final volume in both mixtures.

### 2.4 Antagonist drugs

To assess recovery from anesthesia, we administered 0.5 mg/kg of Atip (atipamezole, Kyoritsu Seiyaku Co., Tokyo, Japan), an α_2_‐adrenoceptor antagonist. The selected dose of Atip, 0.5 mg/kg, was equivalent to the dose of Med, which has been demonstrated to facilitate adequate recovery from anesthesia in mice.^29^ Atip was diluted in saline to a final volume of 0.01 mL per gram of body weight and administered via subcutaneous injection at the dorsocervical region using a sterile 29‐g needle (Myjector, Terumo Corp., Tokyo, Japan). The timing of antagonist administration (40 min postinduction) was selected based on previous studies showing that MMB anesthesia typically maintains a surgical plane for approximately 30–60 min in mice.^3^, ^17^ Our pilot observations also confirmed that the reflex score remained ≥4 up to 30–40 min after induction across most strains. Therefore, administering Atip at this time point enabled us to assess recovery profile during the transition from stable anesthesia to spontaneous emergence, thereby maximizing the sensitivity to detect protocol differences.

### 2.5 Experimental design

The study was conducted in two distinct phases. The primary experiment aimed to evaluate the efficacy and physiological effects of two anesthetic combinations: MMB and dMMB. In this phase, a total of 72 mice, comprising 18 males and 18 females from each of the ICR, B6, and BALB strains, were randomly and systematically assigned to 12 groups (3 strains × 2 sexes × 2 anesthetic treatments [MMB or dMMB]; *n* = 6 per group). Each group received either MMB or dMMB via subcutaneous injection. After administration, several parameters were recorded, including induction time, duration of surgical anesthesia, righting reflex recovery time, and reflex scores. In addition, physiological indicators, such as heart rate, SpO_2_, and body temperature, were continuously monitored.

**FIGURE 1:**
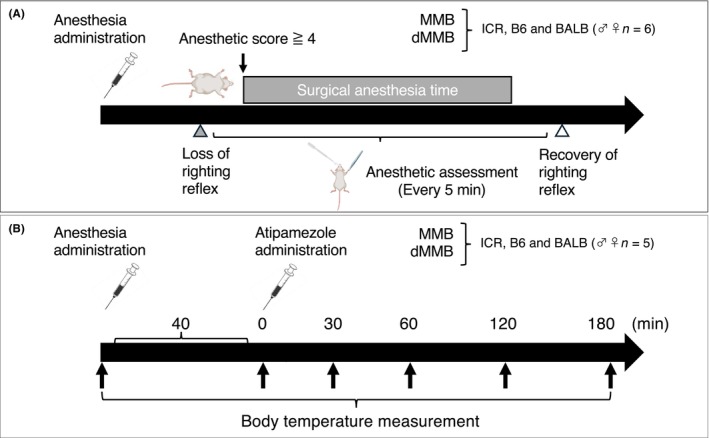
Evaluation of anesthesia and recovery profile in mice administered MMB or dMMB. (A) Experimental design for anesthesia induction, maintenance, and recovery assessment. Mice (ICR, C57BL/6 [B6], and BALB/c [BALB]; both sexes; *n* = 6 per group) were administered either conventional MMB (medetomidine–midazolam–butorphanol) or modified dMMB (dexmedetomidine–midazolam–butorphanol). Righting reflex was assessed to determine the time points of loss and recovery. Anesthetic depth was scored every 5 min, and surgical anesthesia was defined as an anesthetic score ≥4. (B) Body temperature was measured at 30, 60, 120, and 180 min after administration of the antagonist (Atip) in ICR, B6, and BALB mice (*n* = 5 per group) treated either with MMB or dMMB.

The secondary experiment assessed the recovery‐enhancing effects of Atip following MMB or dMMB anesthesia, using a different cohort of mice to ensure independence from the primary experiment and avoid potential carryover effects of repeated anesthetic and antagonist exposure. A total of 60 mice (5 per group) were allocated across 12 groups (3 strains × 2 sexes × 2 anesthetic treatments). Selected mice from each strain received either MMB or dMMB subcutaneously followed by subcutaneous administration of Atip 40 min after anesthetic induction. Recovery parameters, including the time to regain the righting reflex and changes in body temperature, were recorded at 30, 60, 120, and 180 min post‐Atip injection. Rectal temperature was measured at each time point by gently restraining the mice by hand and inserting a thermistor probe into the rectum. To specifically evaluate the natural course of thermoregulatory recovery following anesthesia, external heating was intentionally withheld after Atip administration. The overall experimental design is illustrated in Figure 1A,B.

### 2.6 Animal handling

After recording body weights, anesthetic agents were administered in weight‐adjusted calculated volumes. For subcutaneous injections, mice were manually restrained by gently grasping the loose skin at the back of the neck. This method provides secure immobilization while minimizing stress and enabled consistent injection into the dorsocervical region with a 29‐g needle. To prevent anesthesia‐induced hypothermia, a common issue in small rodents due to their high surface area‐to‐body weight ratio, mice were placed supine on a heating platform built into the PhysioSuite system (Kent Scientific Corporation, Torrington, CT, USA), which was maintained at approximately 37℃. No prewarmed fluids or ophthalmic lubricants were administered, as procedures were of short duration and designed to evaluate the protocols under baseline conditions without supportive interventions. Core body temperature was monitored using a rectal probe, and vital signs, including heart rate and SpO_2_, were measured every 10 min via a sensor attached to the hind limb.

### 2.7 Anesthetic assessment

Time to loss of righting reflex (LORR) was evaluated by continuous observation performed by an investigator blinded to treatment, who gently placed each mouse in a supine position once immobile and recorded the time until all attempts to regain the original posture had ceased. In addition, nociceptive reflex responses were evaluated at 5‐min intervals until the righting reflex returned, following a previously established method.^3^ The nociceptive reflexes were assessed in the following order to minimize variability and stress: (1) righting reflex, (2) tail pinch reflex, (3) pedal withdrawal reflexes of the forelimbs and hind limbs, and (4) eyelid reflex. Reflexes were recorded every 5 min throughout anesthesia. An overall anesthetic depth score was then calculated, with a score ≥4 indicating a surgical level of anesthesia, as previously described. Paw withdrawal reflexes were tested in both forelimbs and hind limbs to provide a comprehensive assessment of anesthetic depth. Because forelimb responses may be less reliable, hind limb withdrawal was regarded as the primary indicator, with forelimb testing used as a supportive measure.^30^ The righting reflex was tested by placing the mouse in a supine position and observing its attempt to return to its original posture. A score of 0 indicated presence and 1 indicated absence of the reflex. The tail pinch reflex was assessed by gently applying nontraumatic forceps to six proximal points along the tail, scored similarly. The pedal withdrawal reflex was evaluated by pinching the interdigital space of each limb using nontraumatic forceps; absence of reflex in both forelimbs and hind limbs was scored as 1. The eyelid reflex was tested by applying a gentle stream of air to the cornea using a Pasteur pipette fitted with a 2‐mL silicone nipple, with responses scored as 0 (present) or 1 (absent). Reflex scores were recorded every 5 min and used directly to assess anesthetic depth. An overall anesthetic depth score was then calculated for each animal, with a score of ≥4 indicating a surgical level of anesthesia, as previously described.

### 2.8 Side effect evaluation

To evaluate potential adverse effects of the anesthetic protocols, two safety parameters were assessed. First, ocular opacity was visually inspected 40 min after anesthetic administration in all animals. Second, blood glucose levels were measured 20 min postinduction by collecting blood via tail incision. A handheld glucometer (FreeStyle Precision Neo, Abbott Japan Co., Ltd., Tokyo, Japan) was used for glucose measurements in accordance with the manufacturer's instructions.

### 2.9 Statistical analysis

**FIGURE 2:**
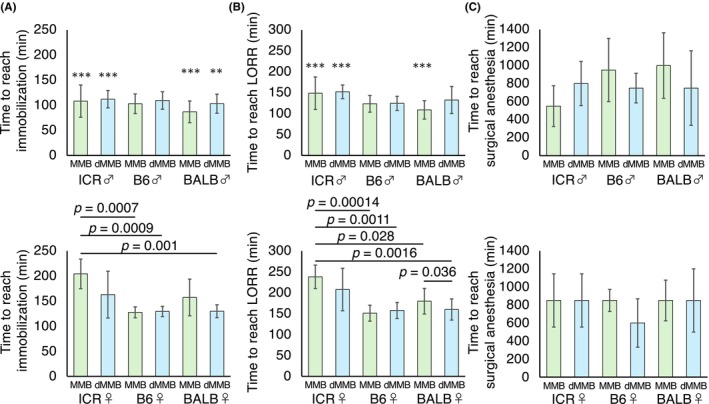
Sex‐ and strain‐dependent differences in anesthesia induction times between MMB and dMMB protocols. Time required to reach (A) immobilization, (B) loss of righting reflex (LORR), and (C) surgical anesthesia in male (top row) and female (bottom row) mice of ICR, C57BL/6 (B6), and BALB/c (BALB) strains following subcutaneous injection of MMB or dMMB. Each bar represents the mean ± standard deviation (SD) (*n* = 6 per group). Asterisks indicate significant sex differences within each strain and anesthetic protocol (unpaired *t*‐test; **p < 0.01, ***p < 0.0001.)

All data are presented as mean ± SD, except for reflex scores, which are presented as median (interquartile range) due to their ordinal scale and non‐normal distribution. For comparisons between two groups involving parametric data—such as detecting sex differences in induction time to immobilization, LORR, and time to reach surgical anesthesia—an unpaired two‐tailed Student's *t*‐test was used. Specifically, sex‐based comparisons within the same strain and anesthetic protocol (e.g., ICR males vs. females under MMB) were analyzed using *t*‐tests and marked with asterisks in Figure 2. For multiple group comparisons of parametric variables, one‐way analysis of variance (ANOVA) followed by Tukey–Kramer honestly significant difference (HSD) post‐hoc tests was applied. All these statistical analyses were conducted using JMP Pro 17 (SAS Institute Inc., Cary, NC, USA). For time course data, such as heart rate, SpO_2_, and body temperature, two‐way repeated‐measures ANOVA was performed using MATLAB (MathWorks, Natick, MA, USA) followed by post‐hoc unpaired *t*‐tests at each time point where appropriate. Reflex scores were analyzed using the Scheirer–Ray–Hare test in R software, version 4.2.1, followed by Bonferroni‐corrected multiple comparisons. A *p*‐value <0.05 was considered statistically significant.

### 2.10 Sample size rationale

The group size was set to *n* = 6 per group based on previous studies of mouse anesthesia that used comparable cohort sizes (approximately six animals per group) to evaluate anesthetic depth and reflex responses. For secondary end points, such as body temperature and cardiorespiratory monitoring, we used *n* = 5 per group, which is also consistent with prior studies of mouse anesthesia and thermoregulation.^30^, ^31^ In addition, our pilot analysis indicated that, for core temperature, an expected effect size (Δ = 0.8℃, SD = 0.6℃; *d* ≈ 1.3) combined with repeated measurements (five time points, within‐subject correlation *ρ* ≈ 0.3–0.4) yields sufficient power (>80%) with *n* = 5–6 animals per group. Taken together, these considerations indicate that the chosen group sizes are adequate to detect biologically meaningful differences while adhering to the 3Rs principle.

## 3 RESULTS

### 3.1 Time‐related indices of anesthesia induction

In this study, no surgical procedures were performed. The term “*surgical anesthesia*” refers exclusively to the depth of anesthesia (a reflex score ≥4) considered adequate for surgical interventions, not to the performance of surgery itself. Across mouse strains and sexes, we evaluated three time‐based parameters: (1) time to immobilization, (2) time to LORR, and (3) time to reach surgical anesthesia. All mice (100%) in both MMB and dMMB groups achieved surgical anesthesia (a reflex score ≥4). The time to reach a surgical plane varied between groups, but all animals eventually achieved adequate anesthetic depth for surgical procedures. No mortality occurred during the experiments. In addition, mice were monitored for 24 h after atipamezole administration and until full recovery. No abnormal clinical signs were observed during this period. Normal feeding, grooming, and locomotor activities were restored within 2–4 h postrecovery.

The time to reach immobilization differed significantly among mouse strains, sexes, and anesthetic protocols (one‐way ANOVA, *p* < 0.0001). When comparing only male mice, no significant differences were observed in immobilization time between MMB and dMMB treatments (one‐way ANOVA showed no significant difference). Conversely, in female mice, immobilization time varied significantly (one‐way ANOVA, *p* = 0.0003). Specifically, female ICR mice treated with MMB exhibited significantly longer immobilization times compared to BALB/c females treated with dMMB, B6 females treated with dMMB, and B6 females treated with MMB. In comparisons between sexes, male mice exhibited significantly shorter induction times to immobilization in the following groups: ICR mice treated with MMB (*p* < 0.0001), BALB/c mice treated with MMB (*p* < 0.0001), ICR mice treated with dMMB (*p* = 0.0009), and BALB/c mice treated with dMMB (*p* = 0.002) (Figure 2A).

The time to LORR also varied significantly among mouse strains, sexes, and anesthetic protocols (one‐way ANOVA, *p* < 0.0001). When comparing only male mice, no significant differences were observed in the time to LORR. Conversely, in female ICR mice treated with MMB, the time to LORR was the longest (238.07 ± 28 s) and was significantly longer than that of all other groups, except for the ICR dMMB group. Specifically, female ICR mice treated with MMB showed a significant difference compared to B6 mice (both MMB and dMMB) and BALB mice (both MMB and dMMB). In contrast, BALB and B6 male mice exhibited the shortest latency, regardless of the anesthetic protocol used. A significant delay in reflex loss was observed in BALB males treated with dMMB compared to those given MMB (*p* < 0.05), whereas other within‐strain comparisons showed no notable differences between MMB and dMMB. When comparing between sexes, females exhibited a significantly longer time to LORR in the following groups: ICR mice treated with MMB (*p* < 0.0001), ICR mice treated with dMMB (*p* < 0.0001), and BALB/c mice treated with MMB (*p* < 0.0001) (Figure 2B).

The time to reach a surgical plane of anesthesia (defined as a reflex score ≥4) did not significantly differ among mouse strains, sexes, and anesthetic protocols. Although BALB males administered MMB required the longest time (1000 ± 363.3 s), and B6 females treated with dMMB reached surgical depth more quickly (600 ± 268.3 s) than those treated with MMB, these differences were not statistically significant. Thus, dMMB and MMB appear similarly effective in achieving surgical anesthesia across the evaluated mouse strains and sexes (Figure 2C).

### 3.2 Duration of surgical anesthesia

**FIGURE 3:**
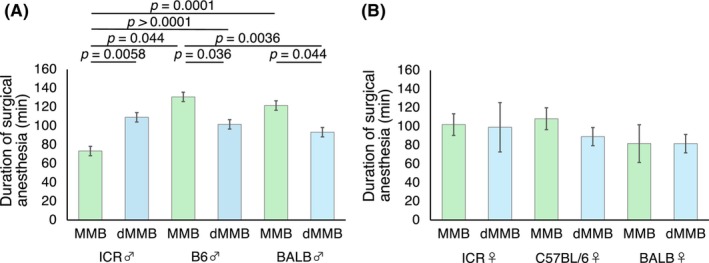
Duration of surgical anesthesia following administration of MMB or dMMB in mice. Surgical anesthesia time (mean ± standard deviation [SD], in min) was compared between MMB and dMMB in male (A) and female (B) mice of three strains: ICR, C57BL/6 (B6), and BALB/c (BALB) (*n* = 6 per group). Surgical anesthesia was defined as the duration during which anesthetic scores remained ≥4. Superscript letters indicate statistically significant differences between groups (*p* < 0.05) based on multiple comparisons.

The duration of surgical anesthesia, defined as the period during which the reflex score remained ≥4, was evaluated separately for male and female mice.^3^ In male mice, there were significant differences in surgical anesthesia duration depending on strain and anesthetic protocol (*p* < 0.05, one‐way ANOVA). ICR males treated with MMB exhibited significantly shorter anesthesia durations compared to several other groups. Tukey–Kramer HSD post‐hoc analysis revealed significant differences between ICR males given MMB and BALB males given MMB (*p* = 0.0001) and B6 males given dMMB (*p* < 0.0001). In contrast, dMMB tended to induce shorter anesthesia durations than MMB in BALB and B6 males, suggesting that Dex may lead to faster recovery in certain genetic backgrounds (Figure 3A).

In female mice, however, no significant differences in anesthesia duration were observed among the groups (*p* > 0.05). The durations were relatively uniform regardless of strain or anesthetic protocol, indicating that the differential effects of dMMB and MMB on surgical anesthesia duration may be more prominent in males than in females (Figure 3B).

**FIGURE 4:**
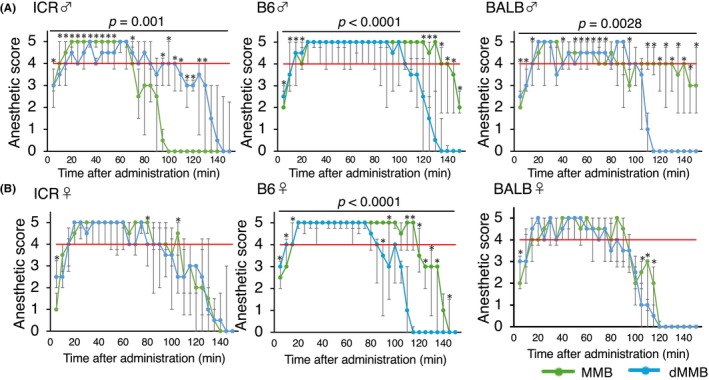
Time course of anesthetic depth following administration of MMB or dMMB in male and female mice. (A) Reflex scores were assessed every 5 min for up to 140 min following administration of MMB or dMMB in male mice of three strains (ICR, C57BL/6 [B6], and BALB/c [BALB]). (B) Same assessment in female mice. Anesthetic scores were evaluated every 5 min for up to 150 min after administration. Scores range from 0 to 5, with higher scores indicating deeper anesthesia. Each point represents the median reflex score at each time point, and the error bars indicate the quartile. Significant differences between the MMB and dMMB groups at each time point were determined using the Scheirer–Ray–Hare test with Bonferroni correction. *p < 0.05.

The time course of anesthetic depth was plotted separately for each sex and strain (Figure 4A,B), with a focus on comparing the two protocols. In male mice (Figure 4A), both MMB and dMMB groups reached surgical depth (a reflex score ≥4) within 15–20 min across all strains. However, the duration and trajectory of anesthetic maintenance differed notably between protocols. In B6 and BALB males, MMB‐treated mice generally maintained high scores longer than those treated with dMMB. However, in ICR males, the MMB group showed a more rapid decline in reflex scores than the dMMB group, indicating earlier recovery under MMB in this strain. In contrast, significant differences indicating faster recovery with dMMB were observed at multiple time points in BALB (*p* = 0.0028) and B6 males (*p* < 0.0001), supporting a consistently shorter anesthetic duration under dMMB in these strains.

In female mice (Figure 4B), the overall anesthetic profiles were more stable than in males, but differences between MMB and dMMB remained evident. MMB‐treated females in B6 and BALB strains showed prolonged anesthetic depth compared to their dMMB counterparts, whereas ICR females showed moderate differences. Reflex scores began to decline earlier in dMMB‐treated groups across all strains. Statistically, among female groups, only B6 mice showed significant differences between MMB and dMMB. No significant differences were observed in ICR or BALB females. These results suggest that dMMB anesthesia may provide a shorter and more predictable anesthetic course compared to MMB, particularly in B6 and BALB mice.

### 3.3 Physiological parameters: Heart rate and SpO_2_

To assess cardiopulmonary stability during anesthesia, heart rate and SpO_2_ were continuously monitored for 60 min following administration of MMB or dMMB. Across all groups, a transient decline in heart rate was observed after induction, with the lowest values typically occurring between 10 and 20 min postadministration, followed by a gradual recovery.

**FIGURE 5:**
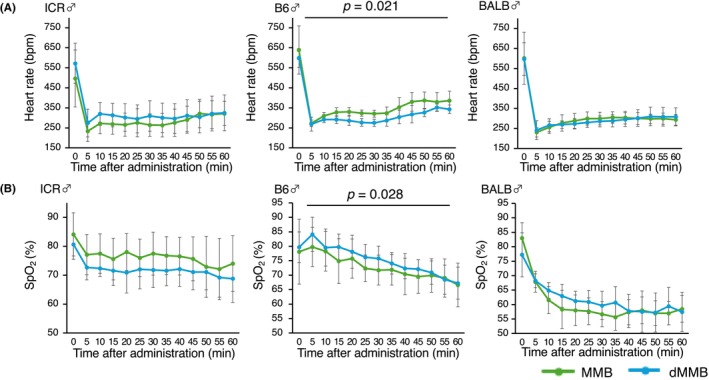
Changes in heart rate and oxygen saturation following administration of MMB or dMMB in male mice. (A) Heart rate (beats per minutes [bpm]) and (B) peripheral oxygen saturation (SpO_2_, %) were monitored in male mice from three strains (ICR, C57BL/6 [B6], and BALB/c [BALB]; *n* = 5 per group) following administration of either MMB or dMMB. Measurements were taken over time to evaluate cardiovascular and respiratory effects under anesthesia. In the B6 strain, dMMB‐treated mice showed a significantly lower heart rate (*p* = 0.021) and a significantly higher SpO_2_ (*p* = 0.028) compared to the MMB group. No significant differences were observed in ICR and BALB/c strains.

In contrast, SpO_2_ levels tended to decline progressively over time, particularly in MMB‐treated mice. This decrease was less pronounced in dMMB‐treated groups in some strains, suggesting a trend toward improved maintenance of oxygenation. In male mice, statistically significant group effects were observed in the B6 strain. Two‐way repeated‐measures ANOVA revealed a significantly lower heart rate (*p* = 0.021) and a significantly higher SpO_2_ (*p* = 0.028) in dMMB‐treated mice compared to those treated with MMB (Figure 5A,B). In BALB/c and ICR males, heart rate and SpO_2_ profiles were similar between groups, and no statistically significant differences were observed.

**FIGURE 6:**
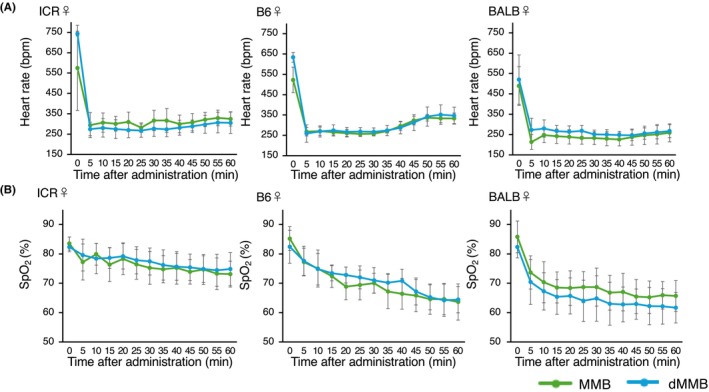
Changes in heart rate and oxygen saturation following administration of MMB or dMMB in female mice. (A) Heart rate (beats per minutes [bpm]) and (B) peripheral oxygen saturation (SpO_2_, %) were monitored in female mice from three strains (ICR, C57BL/6 [B6], and BALB/c [BALB]; *n* = 5 per group) following administration of either MMB or dMMB. Measurements were taken over time to evaluate the physiological effects of anesthesia. No statistically significant differences were observed between the MMB and dMMB groups in either parameter.

In female mice, no statistically significant differences were observed between MMB and dMMB in any strain (BALB, B6, or ICR). Heart rate and SpO_2_ values followed nearly overlapping trajectories, indicating that both protocols produced comparable physiological effects in females (Figure 6A,B).

### 3.4 Side effects: Ocular opacity and blood glucose levels

**FIGURE 7:**
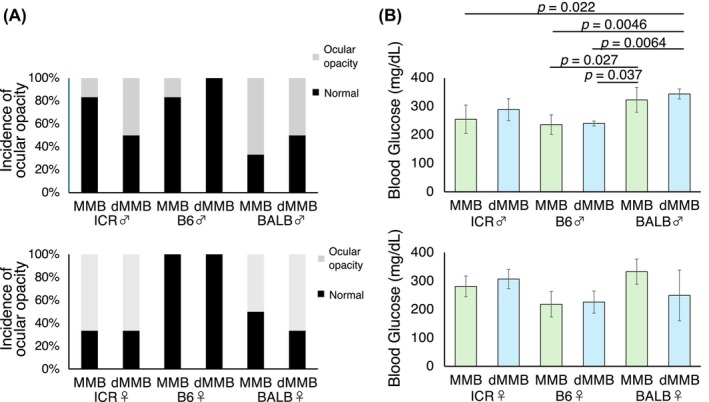
Assessment of side effects following MMB or dMMB anesthesia in male. (A) Incidence of ocular opacity assessed 40 min after anesthesia with MMB or dMMB in three mouse strains (ICR, C57BL/6 [B6], and BALB/c [BALB]). Data represent the percentage of animals showing visible ocular opacity (*n* = 6 per sex per group). (B) Blood glucose levels (mg/dL) measured 20 min after anesthesia in the same groups (*n* = 4 per sex per group).

To assess potential side effects, we evaluated ocular opacity and blood glucose levels following anesthesia. Ocular opacity, potentially associated with transient cataract formation induced by α_2_‐adrenergic agonists,^32^ was observed in a small subset of animals in each group, except for B6 males treated with dMMB and B6 females treated with either MMB or dMMB, in which no opacity was detected. The incidence of unilateral or bilateral opacity did not differ significantly between MMB and dMMB groups in any strain or sex, suggesting that the use of Dex does not alter the risk of this side effect compared to Med (Figure 7A).

Blood glucose levels were also measured to assess metabolic effects, as previous studies have reported that α_2_‐adrenergic agonists can transiently elevate glucose levels in rodents.^33^, ^34^ In male mice, although one‐way ANOVA followed by Tukey–Kramer HSD revealed several significant differences in blood glucose levels across strains and anesthetic protocols, no significant differences were observed between MMB and dMMB within the same strain. This suggests that Dex did not significantly affect glucose levels compared to Med in a strain‐matched manner. In contrast, female mice showed no significant differences in blood glucose levels between MMB and dMMB across all strains. These findings suggest that dMMB may have a mild, strain‐specific effect on glucose metabolism in males (Figure 7B).

### 3.5 Recovery of body temperature after antagonist administration

**FIGURE 8:**
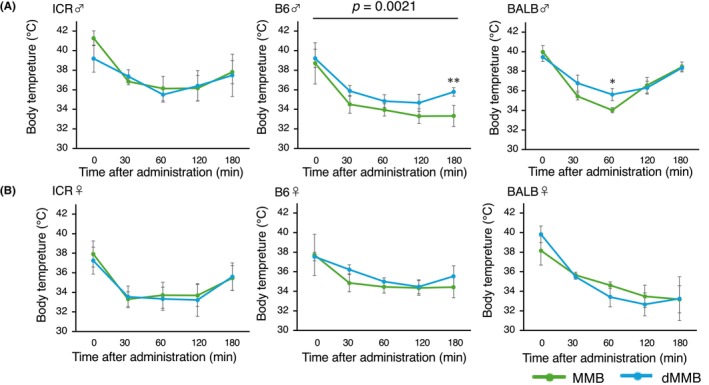
Recovery of body temperature following administration of the antagonist after MMB or dMMB anesthesia in male and female mice. (A) Male and (B) female mice from three strains (ICR, C57BL/6 [B6], and BALB/c [BALB]; *n* = 5 per group) were treated with either MMB or dMMB followed by administration of the antagonist (Atip). Rectal temperature was measured at multiple time points after antagonist injection to assess recovery from anesthesia‐induced hypothermia. Asterisks indicate statistically significant differences between MMB and dMMB groups (*p < 0.05, **p < 0.01). Note that Atip was administered at 0 min, and temperature recovery was tracked relative to this time point.

Rectal temperature was monitored for 180 min following Atip administration to evaluate thermoregulatory recovery after anesthesia. In all strains and both sexes, body temperature decreased during anesthesia and began to return toward baseline after antagonist injection at time 0. In male mice, dMMB‐treated groups generally exhibited faster recovery of body temperature compared to MMB‐treated groups. A significant group effect was detected in B6 males by two‐way repeated‐measures ANOVA (*p* = 0.021), indicating that dMMB facilitated more efficient thermoregulatory recovery in this strain. In BALB males, a significant difference was also observed at 60 min post‐antagonist administration (*p* < 0.05), supporting the superiority of dMMB in promoting temperature normalization (Figure 8A).

In female mice, although a similar trend toward faster recovery was observed in several groups—particularly in the ICR and BALB strains—the differences did not reach statistical significance. Overall, temperature recovery in females was more variable, and the advantage of dMMB was less consistently observed compared to males (Figure 8B). These results suggest that dMMB may enhance thermoregulatory recovery following anesthesia, especially in male mice of certain strains, although the effect appears to be less robust in females.

## 4 DISCUSSION

In this study, we evaluated the efficacy and physiological effects of a modified anesthetic protocol (dMMB), which uses the pharmacologically active dextrorotatory enantiomer Dex instead of conventional triple‐combination anesthesia (MMB), in three mouse strains (ICR, B6, and BALB). By comprehensively assessing anesthesia induction, depth, recovery time, vital signs, and body temperature changes, we found that dMMB provided anesthetic effects comparable to MMB, while showing advantages in terms of body temperature recovery (Table 2).

**TABLE 2:** Summary of strain‐ and sex‐specific differences in anesthetic responses to conventional MMB and modified dMMB protocols in mice.

Parameter	Male	Female	Strain effects	MMB vs dMMB differences
Time to reach immobilization	No significant differences	ICR females (MMB): longest latency	Strain‐ and sex‐dependent	ICR females: MMB > BALB‐dMMB, B6‐dMMB, B6‐MMB
Time to reach LORR	No significant differences	ICR females (MMB): longest latency	Strain‐ and sex‐dependent	BALB females: dMMB slower than MMB
Time to reach surgical anesthesia	No significant differences	No significant differences	–	Comparable between MMB and dMMB
Duration of surgical anesthesia	ICR males: shorter with MMB; BALB & B6 males: shorter with dMMB	No significant differences	Strain‐dependent, esp. males	dMMB → earlier recovery in BALB & B6 males
Heart rate	B6 males: lower with dMMB	No differences	B6‐specific	dMMB < MMB in B6 males
SpO₂	B6 males: higher with dMMB	No differences	B6‐specific	dMMB > MMB in B6 males
Ocular opacity	Occasional in most groups; absent in B6 males (dMMB)	Absent in B6 females (both)	Strain‐dependent	No systematic difference
Blood glucose	Some strain‐specific differences, but no consistent MMB vs dMMB difference	No significant differences	Mild strain effect in males	No clear effect
Temperature recovery (after Atip)	Faster with dMMB in B6 and BALB males	Trend toward faster recovery but NS	Strain‐ and sex‐dependent	dMMB advantage in males, less in females

*Note*: This table summarizes the main findings from the evaluation of conventional MMB (medetomidine–midazolam–butorphanol) and modified dMMB (dexmedetomidine–midazolam–butorphanol) anesthesia across male and female mice of three strains (ICR, C57BL/6, and BALB/c). Parameters include anesthetic induction (time to immobilization, loss of righting reflex [LORR], and surgical anesthesia), maintenance (duration of surgical anesthesia), physiological measures (heart rate and SpO_2_), and side effects (ocular opacity and blood glucose). Recovery of body temperature following atipamezole administration is also listed. Strain‐ and sex‐dependent variations are indicated, along with the relative differences observed between MMB and dMMB protocols.

Abbreviations: LORR, loss of righting reflex; NS, not significant.

First, although some sex‐ and strain‐specific differences were observed—especially in female ICR mice treated with MMB—dMMB showed no systematic delays in anesthesia induction, suggesting comparable onset speed to MMB. Second, clear differences by strain and sex were observed in the duration of surgical anesthesia (defined as the period maintaining a reflex score of 4 or more). In male mice, the ICR strain showed the shortest surgical time under MMB, whereas in BALB and B6 mice, the duration was shorter in the dMMB group than in the MMB group. This suggests that dMMB may result in earlier termination of anesthesia in some genetic backgrounds. In female mice, no significant differences in surgical time were observed across strains or treatments, indicating that the recovery‐promoting effect of dMMB is more prominent in males. This sex difference is consistent with previous studies; for example, Ansah et al. suggested the sex‐related differences in the efficacy of anesthetics and antagonists.^35^

Potential mechanisms for these sex differences include variation in drug metabolism rates, hormonal effects, and thermoregulatory mechanisms. Mid and Bu are metabolized by hepatic CYP enzymes,^36^ which have been shown to exhibit higher activity in females,^37^ suggesting faster drug clearance. In addition, hormonal fluctuations during the estrous cycle are known to affect drug responses in females, potentially altering anesthetic sensitivity in certain phases.^38^

Importantly, although overall cardiopulmonary responses appeared stable, SpO_2_ levels dropped below 60% in some animals, particularly in BALB males under both MMB and dMMB. Such values are indicative of moderate‐to‐severe hypoxemia and suggest that neither protocol ensures adequate oxygenation under ambient air conditions in certain cases. It should be noted, however, that the accuracy of pulse oximetry is generally reliable between 80% and 99% but becomes progressively less accurate at lower saturation levels.^39^ These findings underscore the potential need for supplemental oxygen or mechanical ventilation, especially when applying these anesthetic protocols to prolonged surgical procedures. In our study, all animals were continuously monitored, prepared supportive measures were available if deterioration had progressed, and all mice recovered spontaneously without intervention.

As for side effects, no significant differences in the incidence of ocular opacity—a transient α_2_‐agonist‐associated effect—were observed between protocols, suggesting that Dex does not increase the risk of this adverse event. However, blood glucose analysis revealed significantly higher levels in dMMB‐treated B6 and BALB males, but not in ICR males or females. These findings suggest that Dex may exert strain‐specific effects on glucose metabolism in males, warranting further investigation in metabolic studies.

Dex is the active dextrorotatory enantiomer of Med, whereas Med is a racemic mixture containing both Dex and the pharmacologically inactive levomedetomidine. This compositional difference can influence pharmacodynamics and recovery profiles. A marked difference was observed in thermoregulatory recovery. In male mice, particularly B6 strain, dMMB significantly accelerated body temperature normalization following atipamezole administration. In contrast, female mice showed no significant differences or consistent trends, indicating a possible male‐specific advantage of dMMB in thermoregulation. These results support the use of dMMB for procedures in which rapid thermal recovery is critical. The difference between MMB and dMMB lies in the absence of levomedetomidine in the latter, which may enhance the relative contribution of Dex's α_2_‐adrenoceptor–mediated actions. This pharmacological distinction could partly explain why sex‐related differences in thermoregulatory recovery appeared more prominent in the dMMB groups.

Furthermore, sex differences may also influence the efficacy of Atip in promoting recovery. Prior studies suggest that males tend to exhibit faster anesthesia induction and emergence, whereas females may show more pronounced sympathetic responses to Atip and more vigorous awakening behavior.^40^ In clinical studies in dogs, cats, and horses, Dex has been shown to achieve comparable sedative effects to Med, while enabling faster and more predictable recovery,^41^, ^42^, ^43^, ^44^, ^45^ supporting the broader utility of dMMB.

Body size differences among mouse strains may partly explain the strain‐specific responses observed in this study. ICR mice, being larger outbred animals, may exhibit different pharmacokinetic profiles and thermoregulatory dynamics compared to smaller inbred strains such as C57BL/6. Although anesthetic agents were administered on a mg/kg basis to standardize dosing, inherent differences in body mass and metabolic rate likely contribute to variations in anesthetic depth and recovery. Indeed, strain‐dependent differences in body weight and body composition have been shown to influence metabolic rate in mice,^46^ supporting the notion that such physiological variability could underlie the strain‐specific anesthetic responses observed here. These factors should be considered when extrapolating strain‐specific findings or applying these protocols in broader experimental contexts.

In this study, Atip was administered at the same dose as Med. However, a previous report^24^ demonstrated that a higher dose of Atip significantly enhanced body temperature recovery in MMB‐anesthetized mice. It is therefore plausible that dMMB may confer similar or even superior thermoregulatory benefits under optimized antagonist dosing. Further investigation into the dose‐dependent effects of Atip in the context of dMMB could help refine recovery protocols and enhance its applicability in laboratory settings.

These findings suggest that dMMB may serve as a safe and reliable injectable anesthetic protocol in research settings where ketamine use is restricted, particularly in countries, such as China, with stringent regulatory controls. Notably, Dex formulations are already commercially available in both China and Japan, and their use is expanding in both clinical and basic research. This trend underscores the growing relevance of protocols like dMMB in the near future.

Furthermore, considering the possibility that Med formulations may eventually be discontinued, transitioning to Dex‐based protocols appears inevitable. Therefore, validating the efficacy and safety of dMMB in advance is meaningful for ensuring the long‐term sustainability of experimental animal research.

In conclusion, dMMB represents a promising candidate for a safe, ketamine‐free balanced anesthesia protocol and may serve as a future standard. Beyond demonstrating comparable efficacy to MMB, our findings underscore important strain‐specific and sex‐dependent differences in anesthetic responses, which should be taken into account in experimental design. The occurrence of hypoxemia in some animals highlights the need for supplemental oxygen in prolonged or survival procedures to ensure safety. Clinically, the use of Dex instead of Med provides a practical pathway toward sustainable and regulatory‐compliant protocols, particularly in regions with restricted ketamine availability. Future research should further refine dosing strategies, explore the interaction with antagonist regimens, and assess long‐term outcomes to establish dMMB as a robust and widely applicable anesthetic protocol in laboratory animal science.

## AUTHOR CONTRIBUTIONS

**Masaki Watanabe:** Conceptualization; data curation; formal analysis; writing – original draft. **Ryosuke Nakanishi:** Data curation; formal analysis. **Tomoki Omori:** Data curation; formal analysis. **Takeru Sasaki:** Data curation; formal analysis. **Atsushi Asano:** Conceptualization; methodology. **Nobuya Sasaki:** Conceptualization; project administration; writing – review and editing.

## FUNDING INFORMATION

This research received no external funding.

## CONFLICT OF INTEREST STATEMENT

All authors disclosed no conflict of interest that may directly or indirectly influence the content of the manuscript submitted.

## ETHICS STATEMENT

This study was approved by the Institutional Animal Care and Use Committee (approval no.: 24‐097).

## Data Availability

The data that support the findings of this study are available from the corresponding author upon reasonable request.
